# Supplementary material for: MicroRNAs May Play an Important Role in Sexual Reversal Process of Chinese Soft-Shelled Turtle, Pelodiscus sinensis
Source: Genes (Basel). 2021 Oct 25;12(11):1696. doi: 10.3390/genes12111696 (PMC8620467; doi:10.3390/genes12111696)
Supplement: Supplementary file 1 [file genes-12-01696-s001.zip › genes-1409414-supplementary.pdf]

Supplementary materials

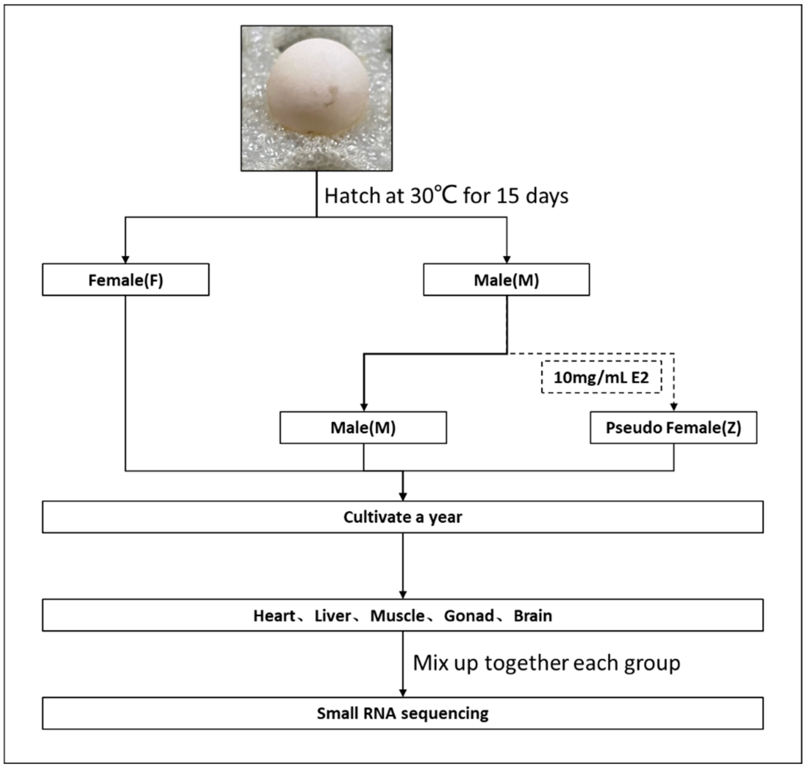

Figure S1: The technical route of exogenous hormones inducing Chinese soft-shelled turtle.

	Group	Total number	female	male	pseudo-female	Sex conversion ratio(%)
Control (Non-injected group)	1	55	29	26	0	0 %
	2	56	25	31	0	0 %
	3	31	15	16	0	0 %
Injected group	1	54	43	11	14	56.00%
	2	55	38	17	13	43.33%
	3	110	79	31	34	52.31%

Figure S2: Statistics of three different types of Chinese soft-shelled turtles. (*i.e.*, female, male, and pseudo-female)

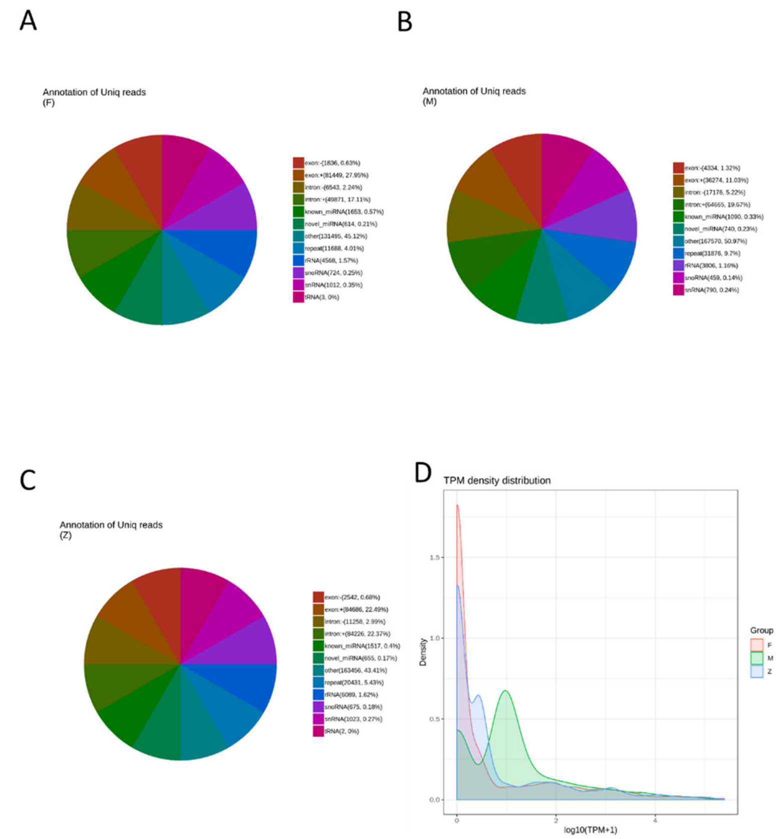

Figure S3: The annotation of unique reads and TPM density distribution of males, females and pseudo-females. (A)The annotation of unique reads of female individuals. (B) The annotation of unique reads of male individuals. (C) The annotation of unique reads of pseudo female individuals. (D)The TPM density distribution of three genetic genders.

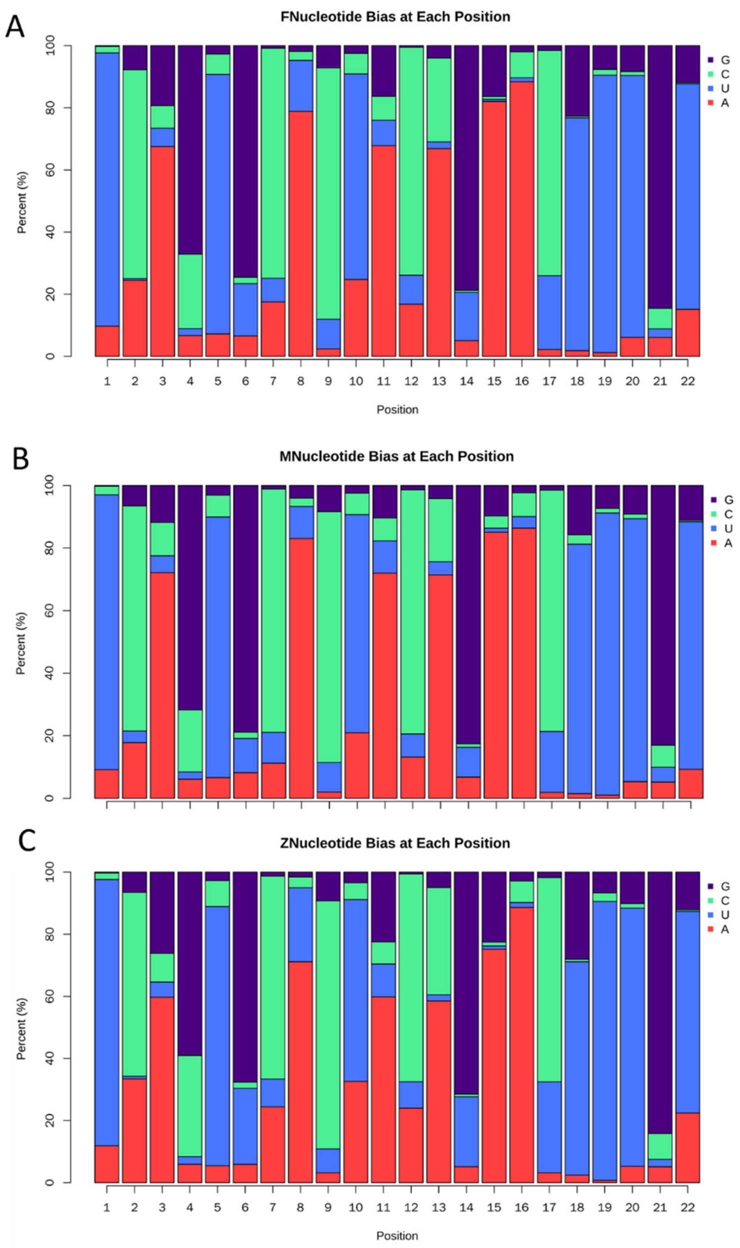

Figure S4: The nucleotide bias at each position of males, females and pseudo-females. (A) The nucleotide bias at each position of female individuals. (B) The nucleotide bias at each position of female individuals. (C) The nucleotide bias at each position of pseudo female individuals.

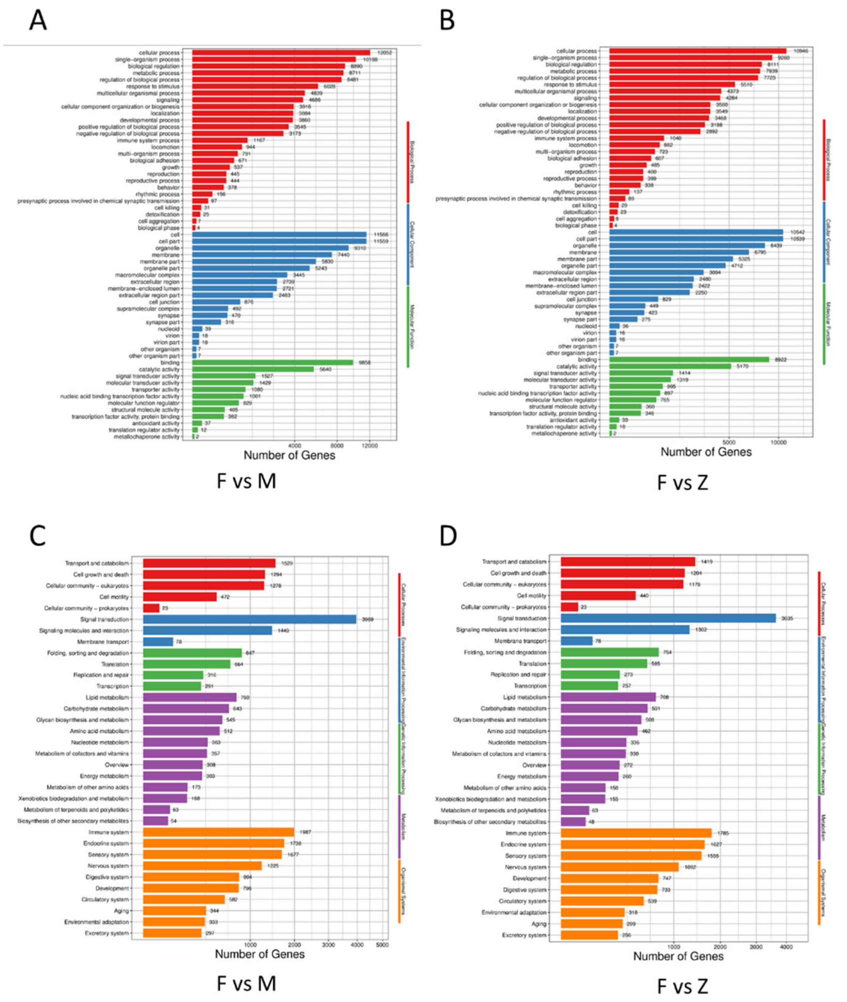

Figure S5: The GO and KEGG enrichment analysis between the female individuals and the other two genetic genders. (A) The GO enrichment analysis between the female individuals and male individuals. (B) The GO enrichment analysis between the female individuals and pseudo female individuals. (C) The KEGG enrichment analysis between the female individuals and male individuals. (D) The KEGG enrichment analysis between the female individuals and pseudo female individuals.
